# Supplementary material for: Impact of deep learning model uncertainty on manual corrections to MRI‐based auto‐segmentation in prostate cancer radiotherapy
Source: J Appl Clin Med Phys. 2025 Aug 24;26(9):e70221. doi: 10.1002/acm2.70221 (PMC12375281; doi:10.1002/acm2.70221)
Supplement: Supplementary file 1 — Supporting Information [file ACM2-26-e70221-s003.pdf]

Supplementary material

Study setup

Internal documentation and methods used in Eclipse during the study are available in the file "Supplementary_Data import and export instructions v2_public.pdf".

Oncologist organ rating study raw data

All oncologist raw data ratings for each question and each subject can be found in the file "Supplementary_ObserverRawData_v2.zip". This zip file contains a copy of the Google Docs document each oncologist used in the study for step 1 and step 2.

Oncologist organ ratings

Table S1. The most common rating was reported for each question/organ, for each oncologist in step 1 and step 2, followed by the highest and lowest in brackets. The number in parentheses following the rating letter is the frequency.

Oncologist	Q1		Q2		Q3 CTV		Q3 Rectum	
	Step 1	Step 2	Step 1	Step 2	Step 1	Step 2	Step 1	Step 2
obsB	B (22)<br>[B-C]	C (18)<br>[B-C]	B (17)<br>[A-C]	B (22)<br>[A-C]	a (21)<br>[a-c]	b (18)<br>[a-b]	a (23)<br>[a-b]	a (21)<br>[a-c]
obsC	B (24)<br>[A-C]	B (15)<br>[A-C]	B (22)<br>[A-C]	B (19)<br>[A-C]	a (19)<br>[a-c]	b (21)<br>[a-b]	a (33)<br>[a-c]	a (29)<br>[a-c]
obsD	B (30)<br>[A-D]	B (32)<br>[A-C]	B (28)<br>[B-C]	B (19)<br>[B-D]	b (23)<br>[a-c]	b (29)<br>[a-c]	a (25)<br>[a-b]	a (19)<br>[a-c]
obsE	A (17)<br>[A-C]	A (26)<br>[A-C]	A (27)<br>[A-C]	A (24)<br>[A-C]	a (29)<br>[a-b]	a (33)<br>[a-b]	a (30)<br>[a-b]	a (34)<br>[a-b]

Oncologist inter-observer differences

Table S2. The median of the differences, i.e., median(step 1-step 2), ± 1 std [minimum, maximum] (p-value) for multiple metrics for the prostate structure for all observers individually. Step 1 and step 2 data were calculated with respect to the reference nnUNet structure. Wilcoxon Signed-Rank Test was used to determine statistical significance for each observer. For “All oncologists as a group”, all data from step 1 and step 2 have been pooled for all observers. A Fligner-Killeen test for homogeneity of variances across all the observers was used to determine if the inter-observer differences between step 1 and step 2 was statistically significant. The difference in variance var(step 1)-var(step 2) is reported as varDiff after the p-value in “Inter-observer difference”. ***** and bold font represent statistically significant results.

	DSC	Surface DSC	HD (mm)	HD95 (mm)	Volume difference (cm^3)	Average surface distance ref2obs (mm)	Total Added Path length (mm)
obsB	**-0.01±0.01 [-0.07, 0.02] (p=0.00) ***	**-0.02±0.05 [-0.24, 0.06] (p=0.00) ***	**0.17±0.86 [-0.57, 2.86] (p=0.00) ***	**0.27±0.55 [-0.94, 1.45] (p=0.00) ***	**0.49±1.12 [-1.59, 5.29] (p=0.00) ***	**0.05±0.15 [-0.17, 0.78] (p=0.00) ***	**33.75±124.32 [-88.13, 623.50] (p=0.00) ***
obsC	-0.00±0.02 [-0.04, 0.03] (p=0.31)	-0.00±0.05 [-0.09, 0.10] (p=0.49)	0.00±1.97 [-3.53, 7.46] (p=0.91)	0.00±0.98 [-1.80, 2.10] (p=0.26)	0.00±1.11 [-3.30, 2.28] (p=0.79)	0.00±0.11 [-0.27, 0.23] (p=0.49)	0.00±99.15 [-186.11, 206.27] (p=0.67)
obsD	**-0.01±0.01 [-0.03, 0.03] (p=0.00) ***	**-0.02±0.05 [-0.13, 0.10] (p=0.00) ***	0.34±1.56 [-5.00, 4.16] (p=0.08)	**0.40±0.83 [-2.10, 1.88] (p=0.01) ***	**-0.61±1.04 [-4.52, 1.14] (p=0.00) ***	**0.04±0.10 [-0.24, 0.32] (p=0.00) ***	**23.44±98.35 [-254.56, 289.25] (p=0.00) ***
obsE	**-0.02±0.02 [-0.07, 0.01] (p=0.00) ***	**-0.07±0.06 [-0.22, 0.01] (p=0.00) ***	**1.20±1.84 [-2.50, 5.00] (p=0.00) ***	**1.48±1.00 [-0.47, 3.13] (p=0.00) ***	0.04±2.20 [-5.17, 4.61] (p=0.88)	**0.17±0.17 [-0.04, 0.53] (p=0.00) ***	**171.11±140.79 [-21.10, 596.31] (p=0.00) ***
All oncologists as a group	**-0.01±0.02 [-0.07, 0.03] (p=0.00) ***	**-0.03±0.06 [-0.24, 0.10] (p=0.00) ***	**0.35±1.73 [-5.00, 7.46] (p=0.00) ***	**0.40±0.99 [-2.10, 3.13] (p=0.00) ***	0.01±1.54 [-5.17, 5.29] (p=0.89)	**0.06±0.15 [-0.27, 0.78] (p=0.00) ***	**36.10±131.11 [-254.56, 623.50] (p=0.00) ***
Inter-observer difference	**p=0.00*, varDiff=0.00**	**p=0.00*, varDiff=0.00**	p=0.08, varDiff=0.05	p=0.21, varDiff=-0.06	**p=0.00*, varDiff=3.64**	**p=0.00*, varDiff=0.02**	**p=0.00*, varDiff=14649.08**

Table S3. Median of the differences, i.e. median(step 1-step 2), ± 1 std [minimum, maximum] (p-value) for multiple metrics for the rectum structure for all observers individually. Step 1 and step 2 data were calculated against the reference nnUNet structure. Wilcoxon Signed-Rank test was used to determine statistical significance for each observer. For "All oncologists as a group", all data from step 1 and step 2 have been pooled for all observers. A Fligner-Killeen test for homogeneity of variances across all the observers was used to determine if the inter-observer differences between step 1 and step 2 were statistically significant. The difference in variance var(step 1)-var(step 2) is reported as varDiff after the p-value in "Inter-observer difference". ***** and bold font represent statistically significant results.

	DSC	SurfaceDSC	HD (mm)	HD95 (mm)	Volume difference (cm^3)	Average surface distance ref2obs (mm)	Total Added Path length (mm)
obsB	-0.00+/-0.02 [-0.10, 0.02] (p=0.05)	-0.01+/-0.03 [-0.14, 0.05] (p=0.09)	0.04+/-4.16 [-4.48, 17.95] (p=0.37)	0.00+/-3.22 [-3.95, 17.45] (p=0.24)	**-0.91+/-2.49 [-13.11, 1.58] (p=0.00) ***	0.00+/-0.15 [-0.61, 0.20] (p=0.52)	0.00+/-68.78 [-218.46, 131.26] (p=0.27)
obsC	0.00+/-0.02 [-0.05, 0.05] (p=0.10)	0.00+/-0.03 [-0.07, 0.06] (p=0.21)	0.00+/-4.87 [-21.79, 8.53] (p=0.23)	**0.00+/-4.21 [-19.71, 7.50] (p=0.03) ***	**-0.61+/-2.39 [-6.75, 6.55] (p=0.04) ***	**-0.08+/-0.65 [-3.16, 0.98] (p=0.01) ***	-22.50+/-141.32 [-438.80, 414.89] (p=0.07)
obsD	0.00+/-0.02 [-0.02, 0.09] (p=0.54)	0.00+/-0.03 [-0.03, 0.15] (p=0.38)	0.00+/-5.78 [-22.72, 9.39] (p=0.44)	0.00+/-4.45 [-15.00, 9.00] (p=0.14)	-0.20+/-2.69 [-12.94, 3.03] (p=0.24)	-0.01+/-0.58 [-2.03, 1.45] (p=0.10)	-7.50+/-156.86 [-766.02, 146.27] (p=0.20)
obsE	-0.00+/-0.03 [-0.12, 0.06] (p=0.05)	-0.00+/-0.04 [-0.17, 0.06] (p=0.06)	0.19+/-10.07 [-7.24, 43.43] (p=0.12)	**0.00+/-7.53 [-2.50, 32.92] (p=0.01) ***	-0.02+/-7.81 [-22.13, 38.89] (p=0.19)	**0.01+/-0.85 [-0.03, 5.15] (p=0.00) ***	**6.56+/-43.82 [-20.16, 224.56] (p=0.00) ***
All oncologists as a group	0.00+/-0.02 [-0.12, 0.09] (p=0.48)	0.00+/-0.03 [-0.17, 0.15] (p=0.46)	0.00+/-6.90 [-22.72, 43.43] (p=0.84)	0.00+/-5.39 [-19.71, 32.92] (p=0.73)	**-0.51+/-4.50 [-22.13, 38.89] (p=0.00) ***	0.00+/-0.63 [-3.16, 5.15] (p=0.09)	0.00+/-115.75 [-766.02, 414.89] (p=0.21)
Inter-observer difference	p=0.79, varDiff=0.00	p=0.65, varDiff=-0.00	p=0.98, varDiff=-2.41	p=0.18, varDiff=2.37	p=0.76, varDiff=-2.92	p=0.17, varDiff=-0.19	p=0.22, varDiff=-12575.43

Inter-observer differences

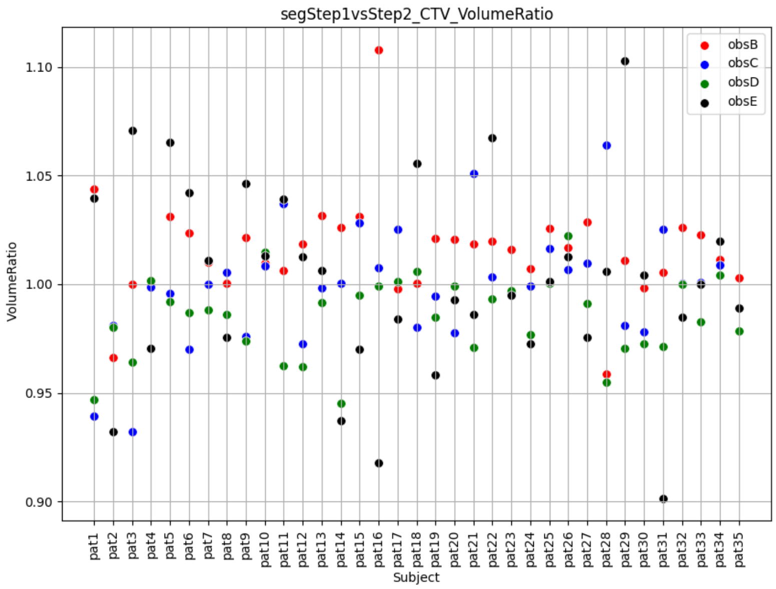

Fig.S1. Volume ratio step 2/step 1 among all patients for all observers in the test dataset for CTV.

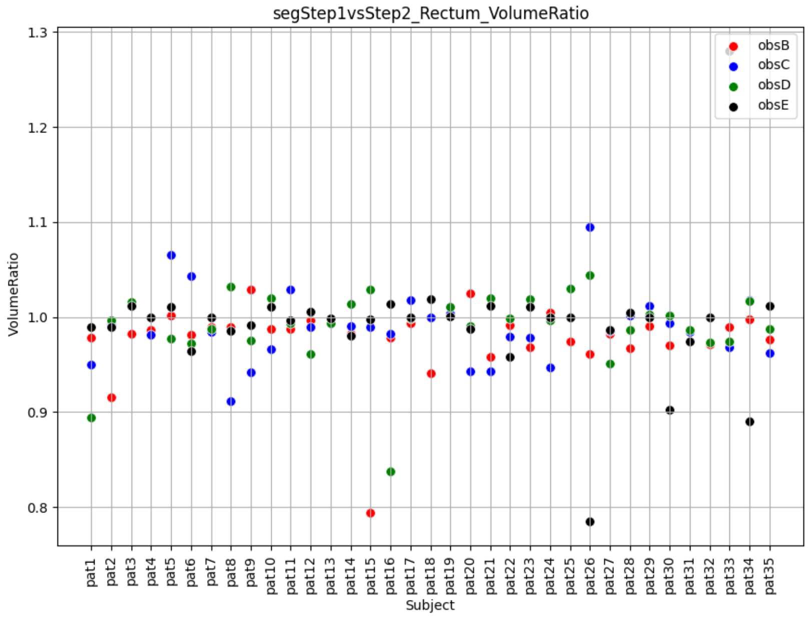

Fig.S2. Volume ratio step 2/step 1 among all patients for all observers in the test dataset for the rectum.

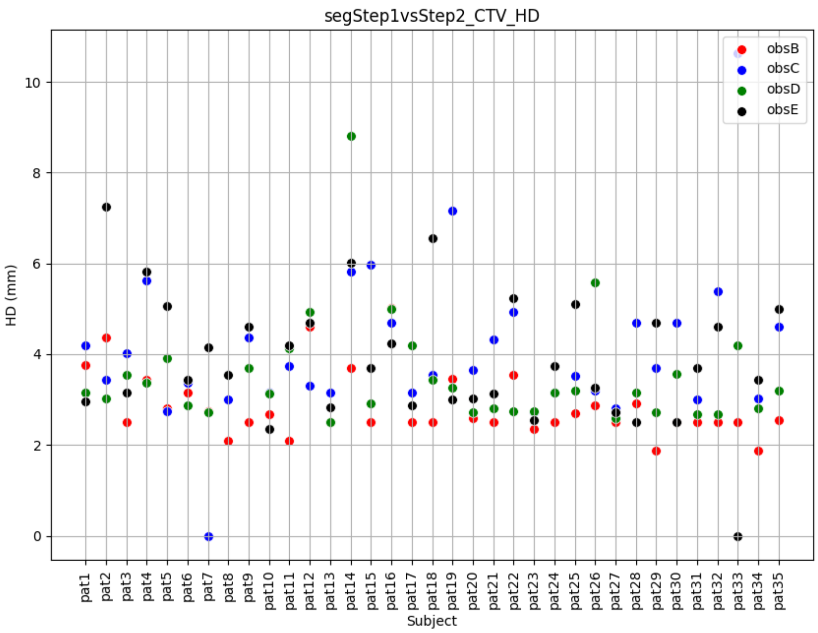

Fig.S3. Hausdorff distance between step 2 and step 1 among all patients for all observers in the test dataset for the prostate.

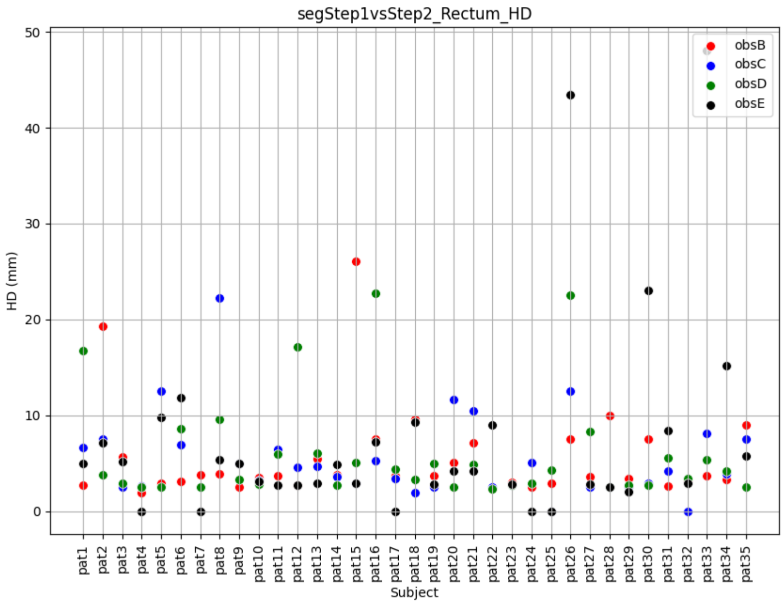

Fig.S4. Hausdorff distance between step 2 and step 1 among all patients for all observers in the test dataset for the rectum.

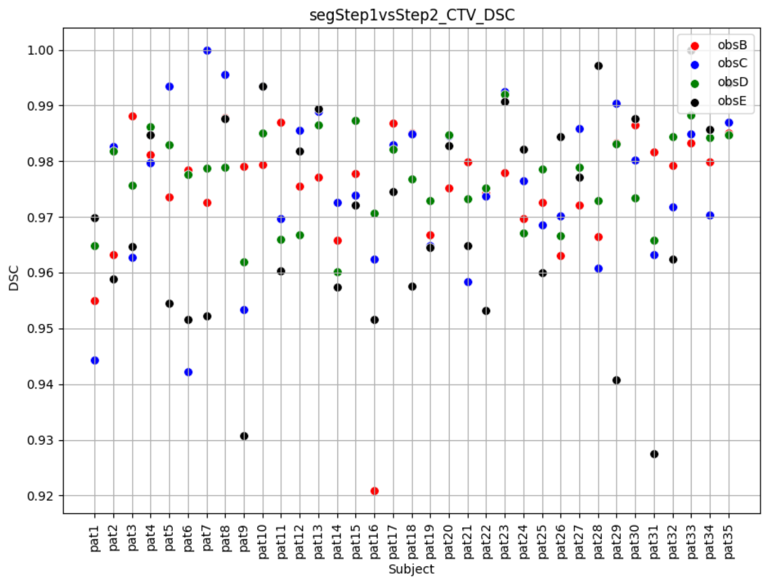

Fig.S5. Dice and between step 1 and step 2 for the prostate.

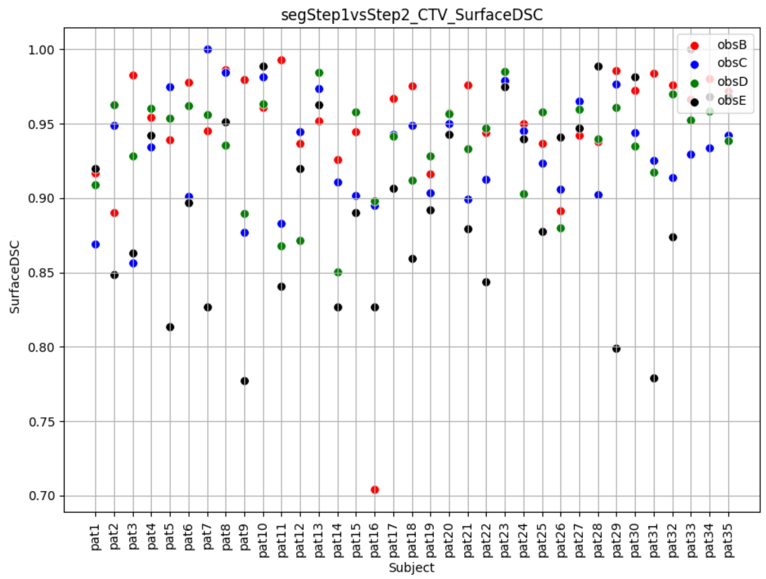

Fig.S6. Surface Dice between step 1 and step 2 for the prostate.

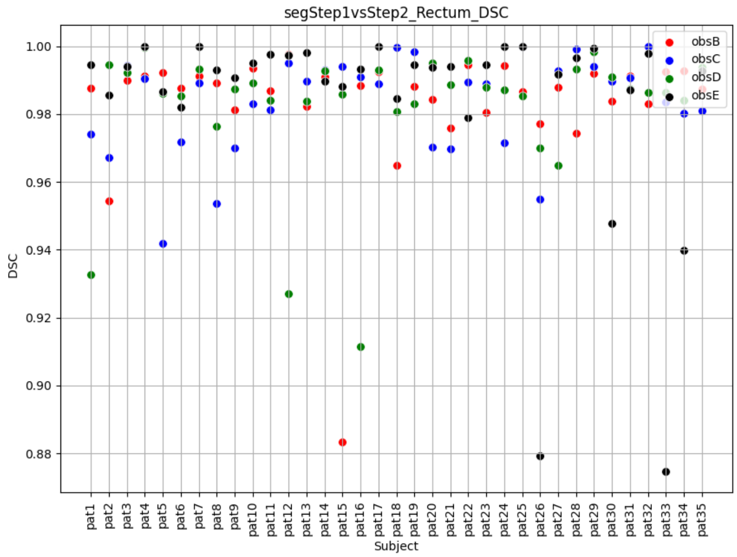

Fig.S7. Dice between step 1 and step 2 for the rectum.

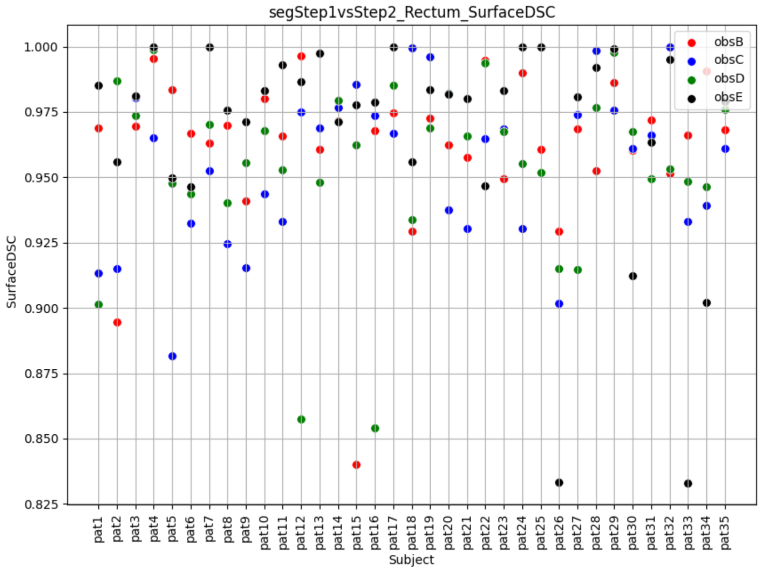

Fig.S8. Surface Dice between step 1 and step 2 for the rectum.

Outlier analysis

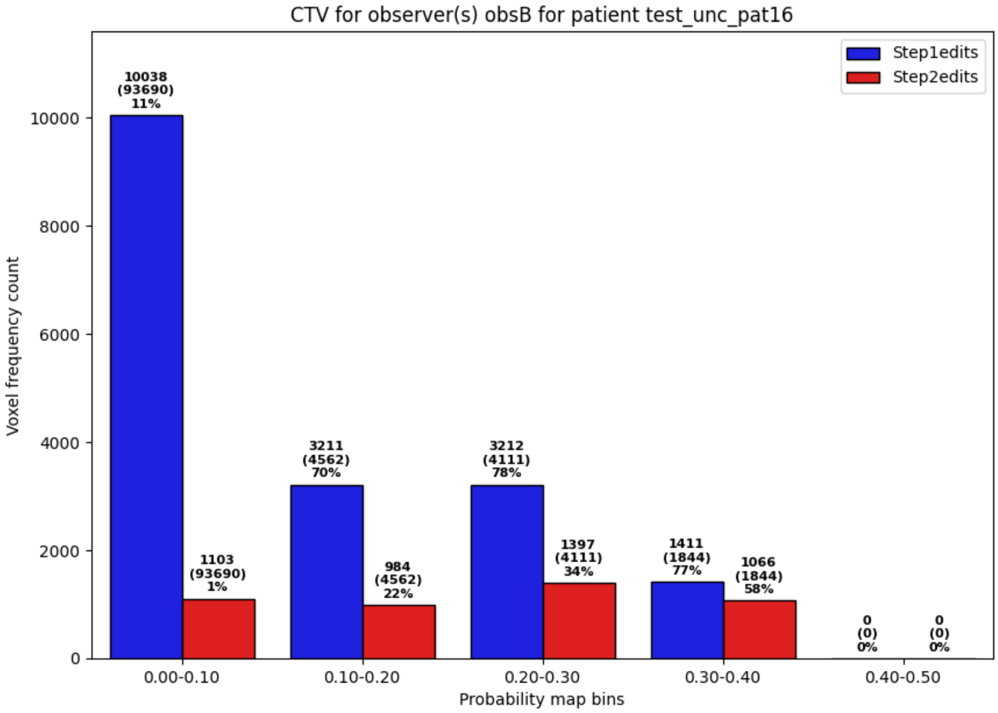

Fig.S9. Histogram over changed voxels for the prostate outlier patient. Fewer voxels were changed in step 2. Above each bar is the number of changed voxels, and in parentheses is the total number of voxels existing within that probability range. The ratio is calculated as a percentage.

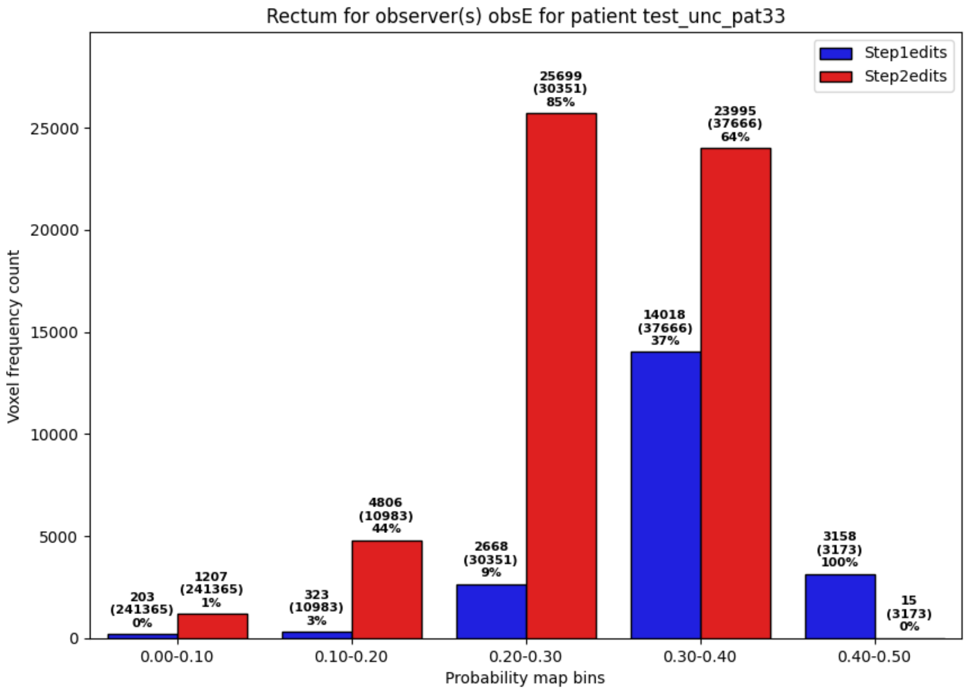

Fig.S10. Histogram over changed voxels for the rectum outlier patient. Above each bar is the number of changed voxels, and in parentheses is the total number of voxels existing within that probability range. The ratio is calculated as a percentage.

Free text answers from oncologists

The free text answer to the question before step 2 is reported below.

Do you think delineation uncertainty information will provide you with useful information?

obsB: ”I am uncertain, but hopefully it will aid in knowing where to look even more carefully.”

obsC: “Yes.”

obsD: “No, I don’t think so.”

obsE: “I think it will help a lot to improve the correctness of my drawing.”

The free text answer to the questions after step 2 is reported below.

1. Do you think delineation uncertainty information has provided you with useful information?

obsB: “Yes, on some occasions the "color" enabled me to discover things I had to edit. At other occasions, when I doubled checked an edit I had made with the color, the color made me realize that an edit I had performed was actually not correct.”

obsC: “Very modest.”

obsD: “I don’t think so. There were no surprises in the areas where there were the most uncertainty (base, ventrally in the apex and so on)”

obsE: ”Yes, I found it very useful.”

2. What was the benefits with delineation uncertainty information?

obsB: “a) Areas with minimal color can be scrolled through fast. b) I can look more careful at areas with much color. c) I can double-check editing, in particular, if it is outside of the color.”

obsC: “To quickly see where to focus more thoroughly.”

obsD: "I don’t see any. In reality, when drawing (planning), we take into account a lot of information that is missing here, especially tumor characteristics (T stage, histopathology report, location of lesions on MRI and PET with risk for EPE and SVI, where cancer was found in biopsies, etc.) but also patient characteristics (age, comorbidities, erectile function, ...). Delineation uncertainty does not, of course, replace this."

obsE: “I was able to determine the correctness of the drawing faster and with greater certainty.”

3. What was the drawbacks with delineation uncertainty information?

obsB: “You feel as if the "allowed" area for editing is only within the color. Areas within the color can easily be edited, but for areas outside the color you need to be very sure you are right before you edit.

Unexperienced contourers will rarely/never edit outside the color, which could be problematic it the color is actually wrong.”

obsC: “No obvious but could potentially if incorrect take focus from important areas of interest.”

obsD: “Not really any, except that a few extra seconds were spent looking at it.”

obsE: “I have not experienced any drawback to the information.”

4. What do you think the potential of delineation uncertainty information is?

obsB: “To guide your attention towards areas of color/variation = put your time where it is most needed. This benefit has to be balanced against the risk of not editing incorrect delineations that are outside the uncertainty map.”

obsC: “I think they can be used in different ways in quick adaptive workflows.”

obsD: “Unclear. Perhaps of interest to inexperienced doctors for educational purposes. It could be a complement to the clinical information I mentioned in point 2."

obsE: “It can help with daily work.”
