# Supplementary material for: Impact of deep learning model uncertainty on manual corrections to MRI‐based auto‐segmentation in prostate cancer radiotherapy
Source: J Appl Clin Med Phys. 2025 Aug 24;26(9):e70221. doi: 10.1002/acm2.70221 (PMC12375281; doi:10.1002/acm2.70221)
Supplement: Supplementary file 2 — Supporting Information [file ACM2-26-e70221-s001.pdf]

Data import, edit and export instructions

Headlines in this document are put in the order of things to happen chronologically overall in the study data flow.

However, for **oncologists** these headlines are the essential ones to look into.

Observer study instructions - Step 1
Import of data for oncologist to edit structure **without** uncertainty map.

Observer study instructions - Step 2
Import of data for oncologist to edit structure again **with** uncertainty map.

Import - Step 1 – Physicist step

- Copy data from inference data\exportEclipse to VA_TRANSFER (for example \\XXX.XXX.XXX.XXX\data2\Christian\MRIOnlyData\inference\MRI-only_test_data_obsA\exportEclipse\test_unc_pat3_obsA and \\XXX.XXX.XXX.XXX \VA_TRANSFER\Temp\Temp Jonas CJG\Christian)
- Open External Beam Planning
- Create a new patient in eclipse. test_unc_patX_obsN. Match patient and observer with data. **Last name = ID = test_unc_patX_obsN, no first name.**
- Assign SUS institution. SUS/Lund/SUS.
- Import data by selecting the step 1 folder for the specific patient.

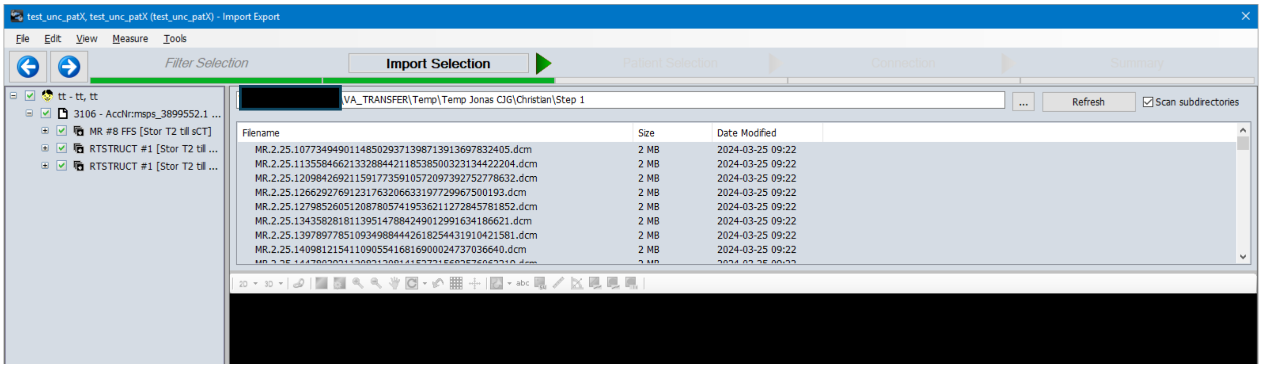

- Browse patients to current selection if not auto selected.

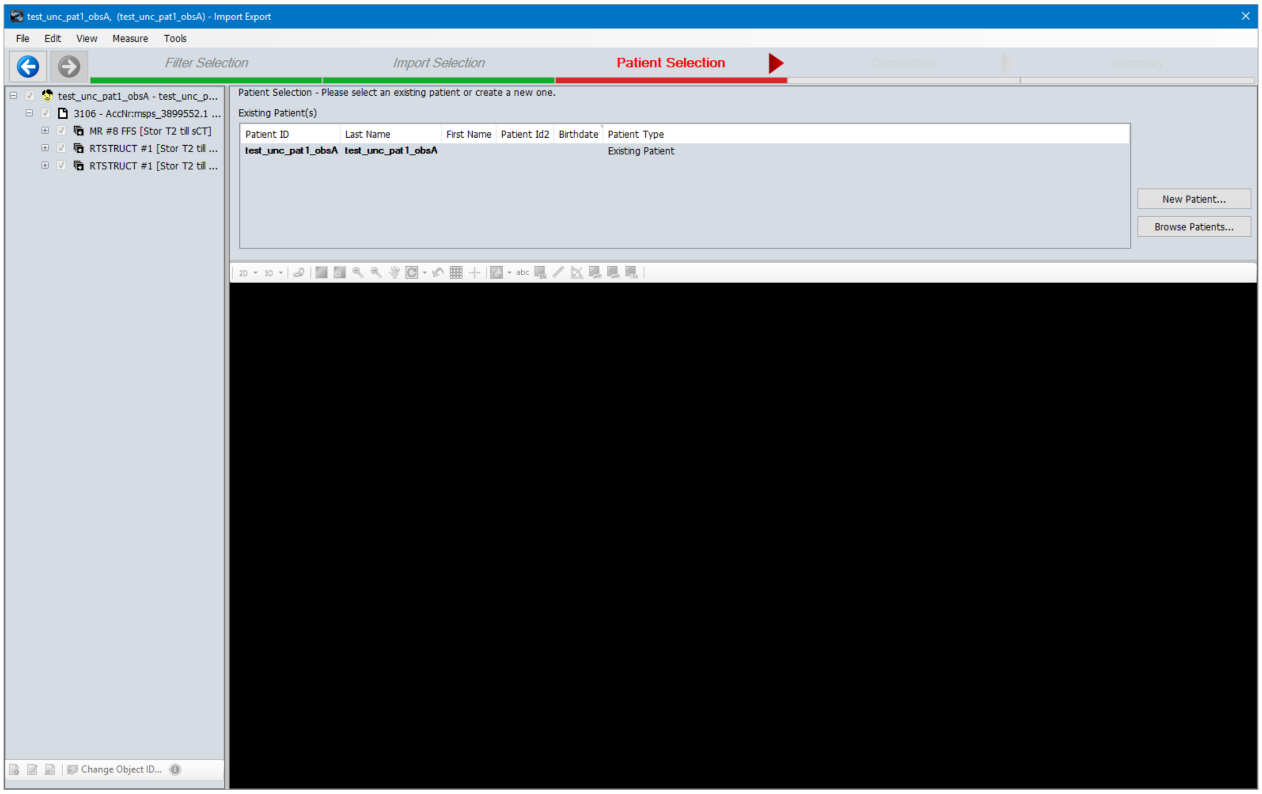

- You will get warnings, that’s ok. Make sure its only warning and not errors. Look at the symbol. Red = error, yellow = warning.

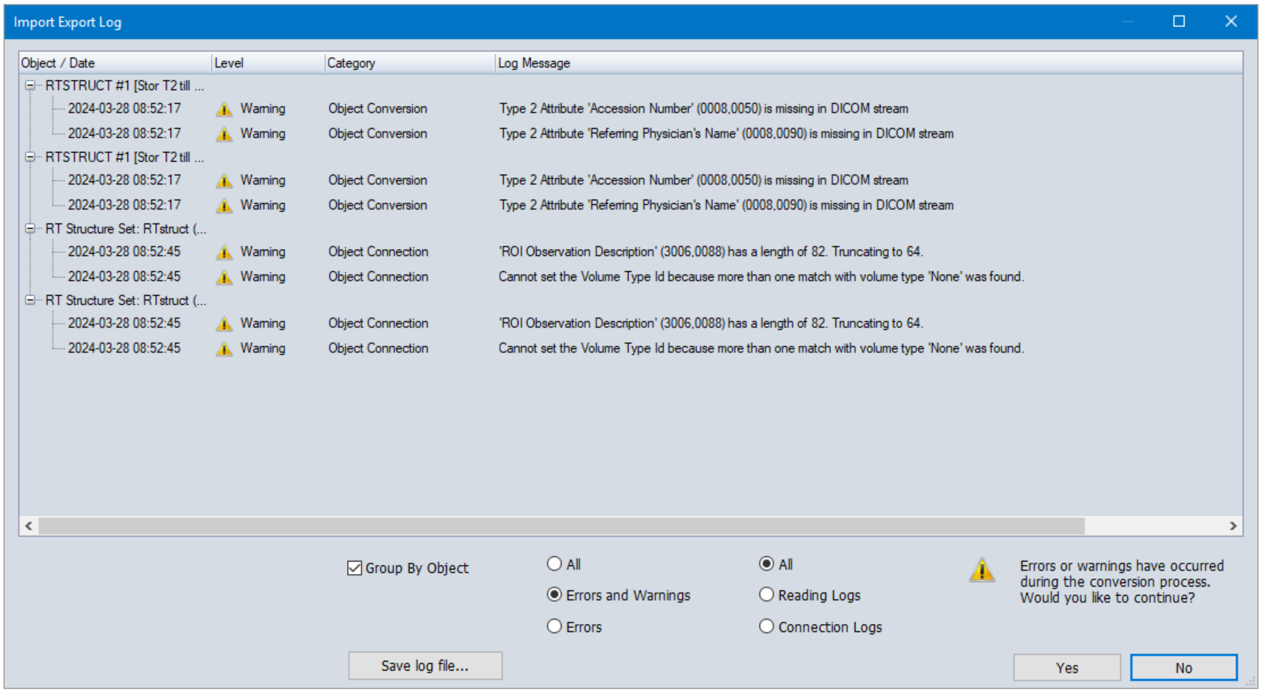

- Results should look like

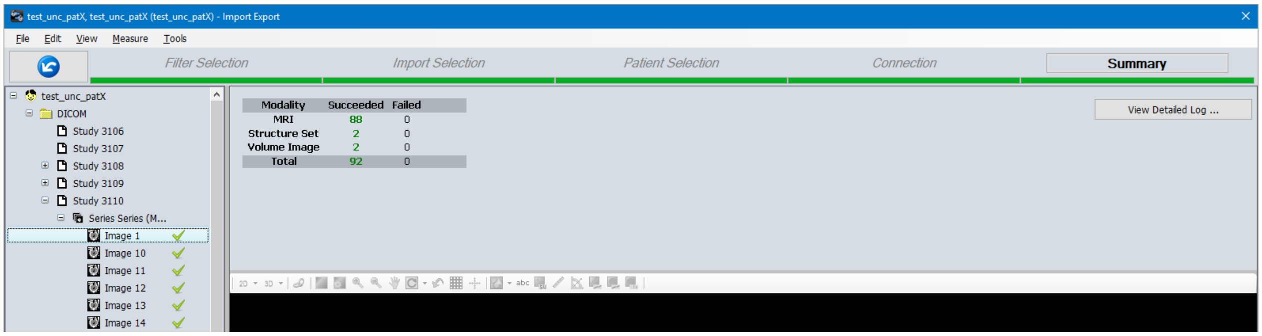

- Rename image volume for CTV to CTV1. (First step). Similar for rectum struct to Rectum1 (First step).

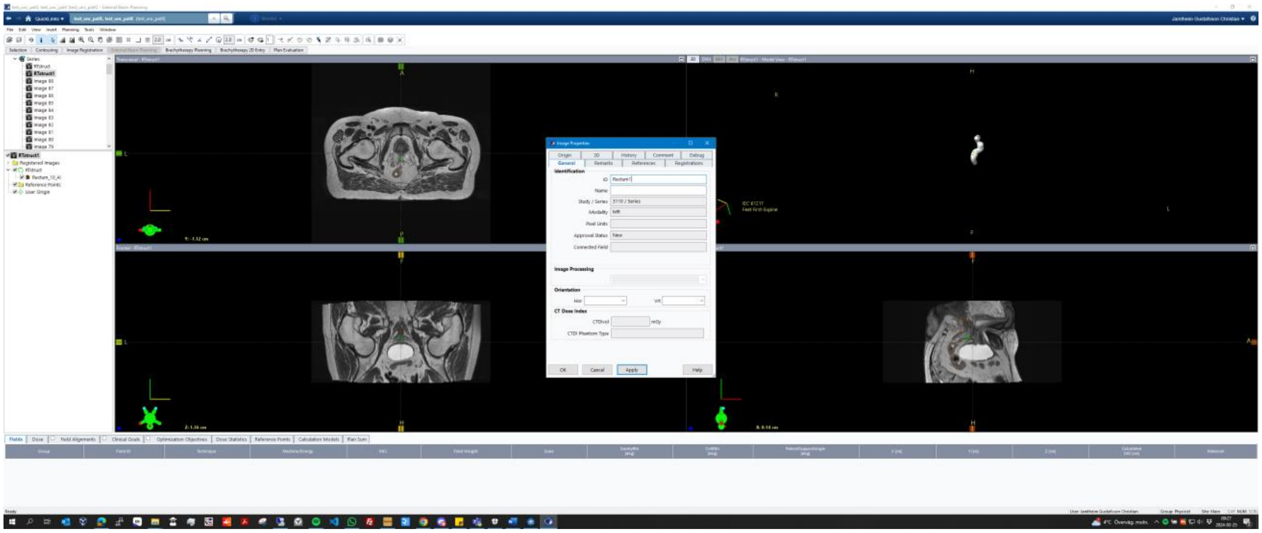

- Save
- Should look like this in contouring

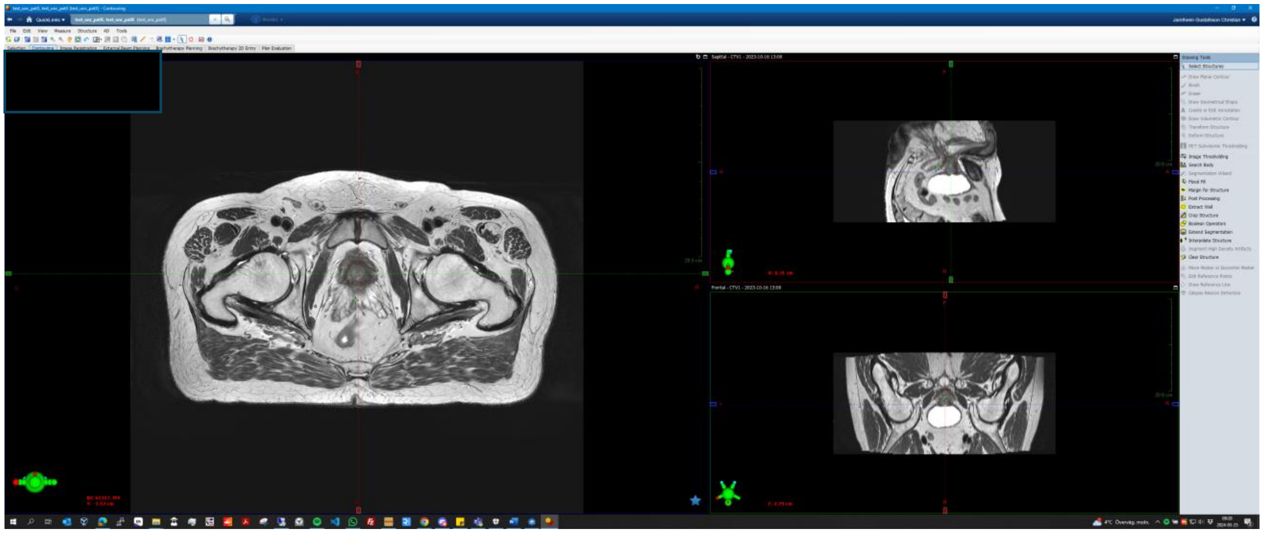

Observer study instructions - Step 1

Aim: Assess the quality of the AI generated structures for prostate CTV and Rectum as OAR and edit them as needed. This is performed without the uncertainty information to create a baseline.

This is performed in the large FOV T2w MRI used for patients with intermediate and high risk prostate cancer treated with MRI-only prostate ultra-hypofractionated radiotherapy (excluding vesicles). Edits of the AI generated structures should be performed in the way as the structures are delineated for clinical MRI only prostate patients. This clinical practice is based on “ESTRO ACROP consensus guideline on CT- and MRI-based target volume delineation for primary radiation therapy of localized prostate cancer”. Radiother Oncol. 2018 Apr;127(1):49-61.

* You are about to assess and edit auto generated structures, remember that all such structures are computer generated and may therefore be incorrect. Edits to the structures will be recorded.

Open the dedicated Google sheet depending on what observer identify you have. It contains assessment questions and a subject matrix to keep track of and record finished subjects.

Link for the Google sheet has been provided to each observer in an email.

Look at the patient matrix in the google sheet. Handle/perform the subjects in the specified order. You will find the full patient pointer at the **subject name + _obsN**). For example, if you are observer B you will find the first patient at “**test_unc_pat17_obsB**”.

Subject	Two structures edited/approved	Q1	Q2	Q3 CTV	Q3 rectum	Estimated time
	Put green color in cell when both done	A/B/C/D	A/B/C/D	a/b/c/d/e	a/b/c/d/e	consumption (min)
test_unc_pat17						
test_unc_pat28						
test_unc_pat23						
test_unc_pat6						
test_unc_pat25						
test_unc_pat30						

- Open selected patient in Contouring. Reorient patient if needed.

- Double check that you loaded the correct patient, as this is observer specific, we recommend copy and pasting the subject name. Subject order in the study is randomized according to the Google sheet list.

- When patient is loaded it looks something like

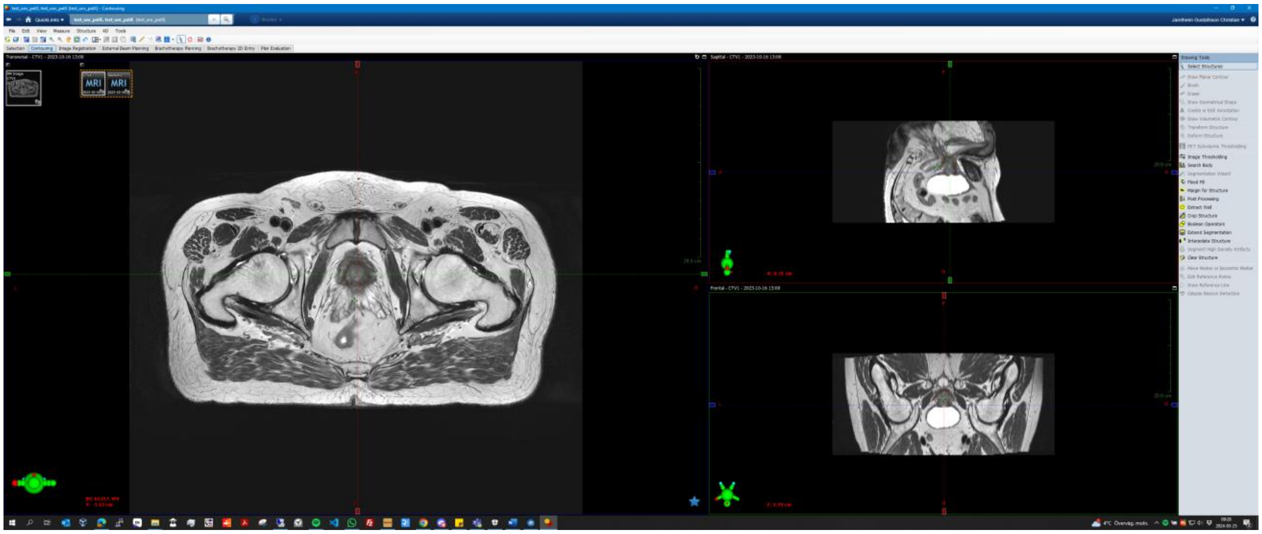

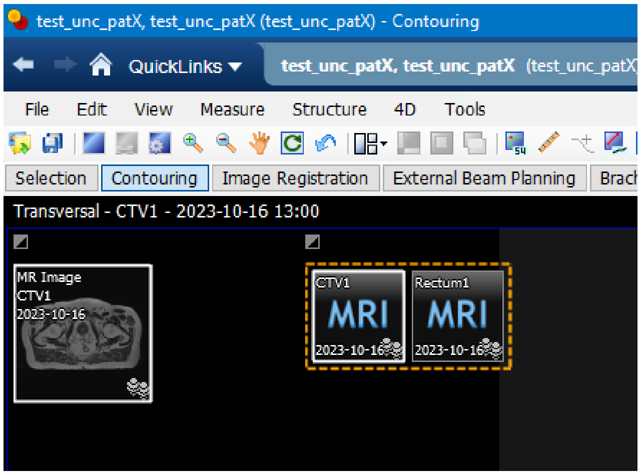

- There are two volumes named CTV1 and Rectum1. Double click one of them as the first one. The volumes are identical but contains the different structures.

- Please review questions in the Google sheet and answer question 1 and 2 by providing your answer in the subject matrix under each specific question (Q1 and Q2). Observe the answer alternatives. **Very small deviations < 1 mm in a slice is not counted for CTV. Corresponding value for rectum is < 2mm.**

- It is important that you have answered the questions Q1 and Q2 before performing stucture edits. Perform your edits (if needed). Perform edits even if they are very small.

- Select the next volume by double clicking it.

For CTV the structure is called CTVT_427_10_AI

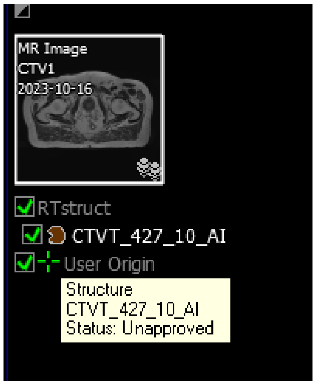

For Rectum the structure is called Rectum_10_AI

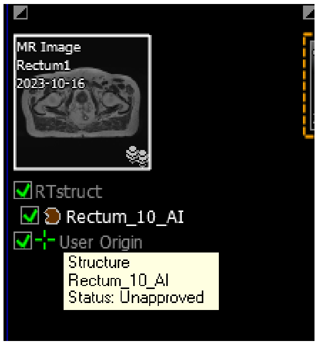

- Answer the question 3 in the Google sheet. Providing your answer in the subject matrix under Q3 CTV and Q3 rectum. Observe the answer alternatives.

- Save patient after edit is finished.

- Mark the patient as complete in the Google sheet by setting a green box in the cell. Make sure you answer all questions and put a green box for all finished tasks.

- Estimate the time it took per patient in minutes and fill in the Google sheet for each subject.

- Repeat the process for the next subject in the Google sheet.

Note: We will let at least 4 weeks pass for the seen information to "wash out" from human memory. This should be counted from the moment when the last structure was edited for the specific oncologist. When this is finished, a new Google sheet for Step 2 will be created with the same questions and similar procedure (but with uncertainty map available). Christian will keep track of time.

Export - Step 1

- Open selected patient for correct observer and patient.

- File/Export/ to MRI only DICOM node.

- Enable everything in tree

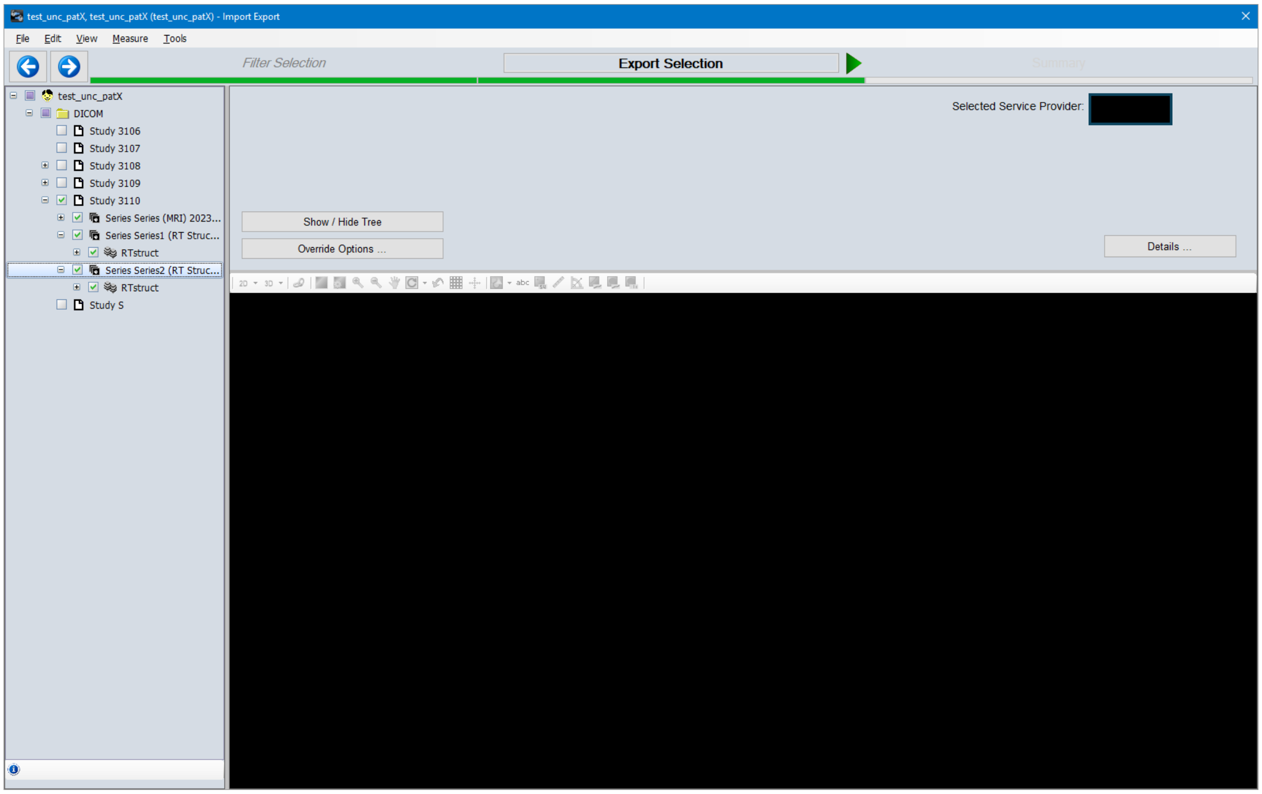

- Check SF archive under RT struct folder. There we will find the edited and exported structures.

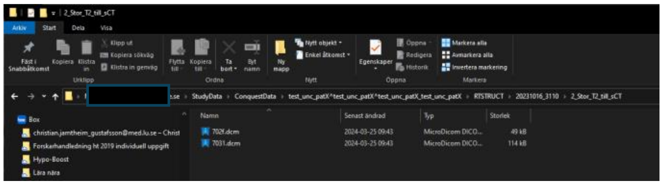

- Copy the data including the MRI. In this case it is [\\XXX.XXX.XXX.XXX\StudyData\ConquestData\test_unc_patX^test_unc_patX^test_unc_patX_test_unc_patX](\\XXX.XXX.XXX.XXX\StudyData\ConquestData\test_unc_patX^test_unc_patX^test_unc_patX_test_unc_patX).
  Move data to archived study folder
  \\XXX.XXX.XXX.XXX\data2\Christian\MRIOnlyData\inference\observerDataEdited
  /obsN/patientX/step1Edits/Rectum1/
  /obsN/patientX/step1Edits/CTV1/

- Delete the CTV1 and Rectum1 structure in Eclipse. Both on RT structure level and volume level.

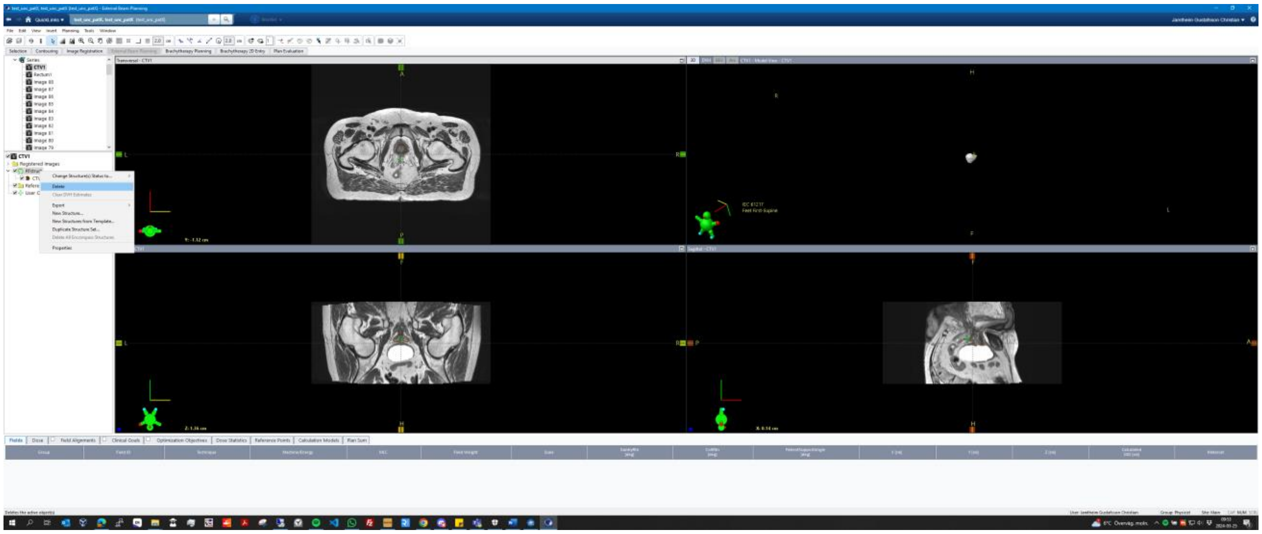

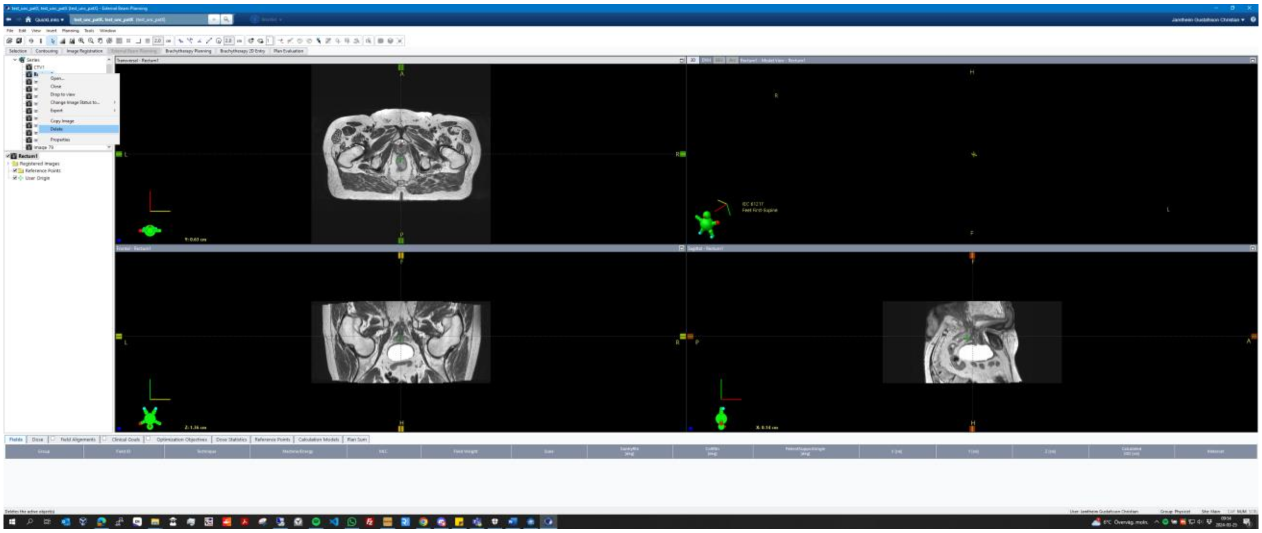

- Save

Import - Step 2 – Physicist step

After the oncologist changing the delineation from Step 1 and data has been exported by Christian it's time to import more data to prepare for Step 2. Remember that Christian has to delete the edited structures after export of Step 1 is finished. It cannot remain in the system for step 2.

- Open selected patient for correct observer and patient according to data.

- Import data and point to step 2 folder for the specific patient. This contains the 2 uncertainty maps and the two copies of the CTV and Rectum structures (having unique UIDs)

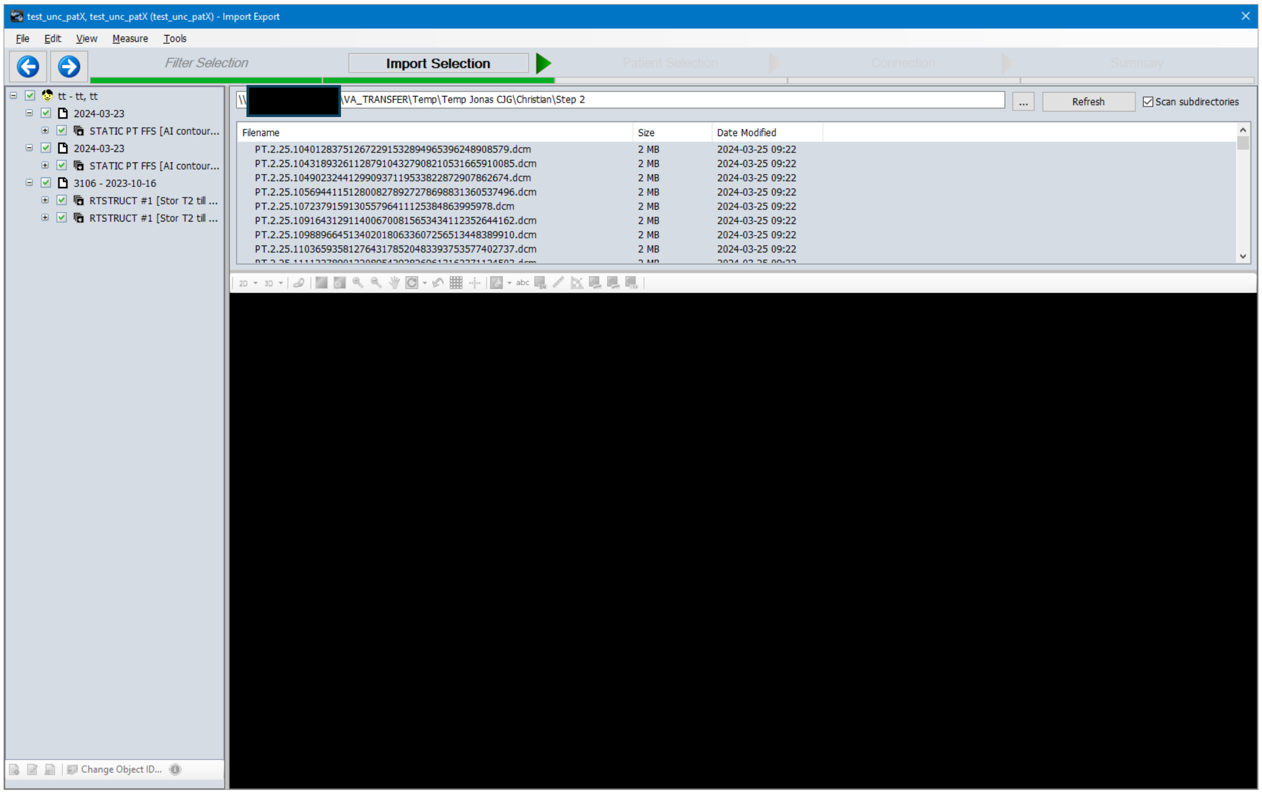

- Browse patients to current selection if not auto matched.

- You will get warnings, that's ok. Make sure symbol is yellow, not red.

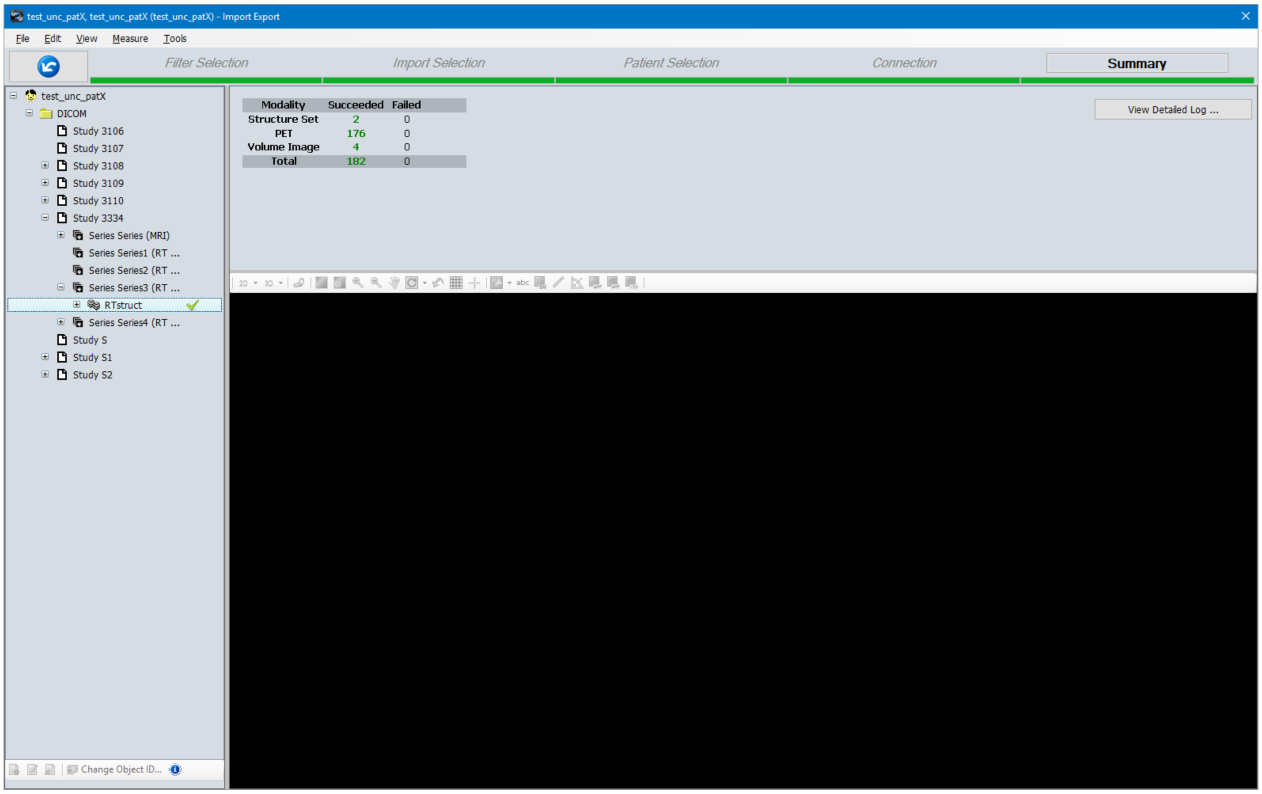

- Rename PET coded uncertainty map for rectum to “unc_rectum2”. For CTV make it “unc_CTV2”.

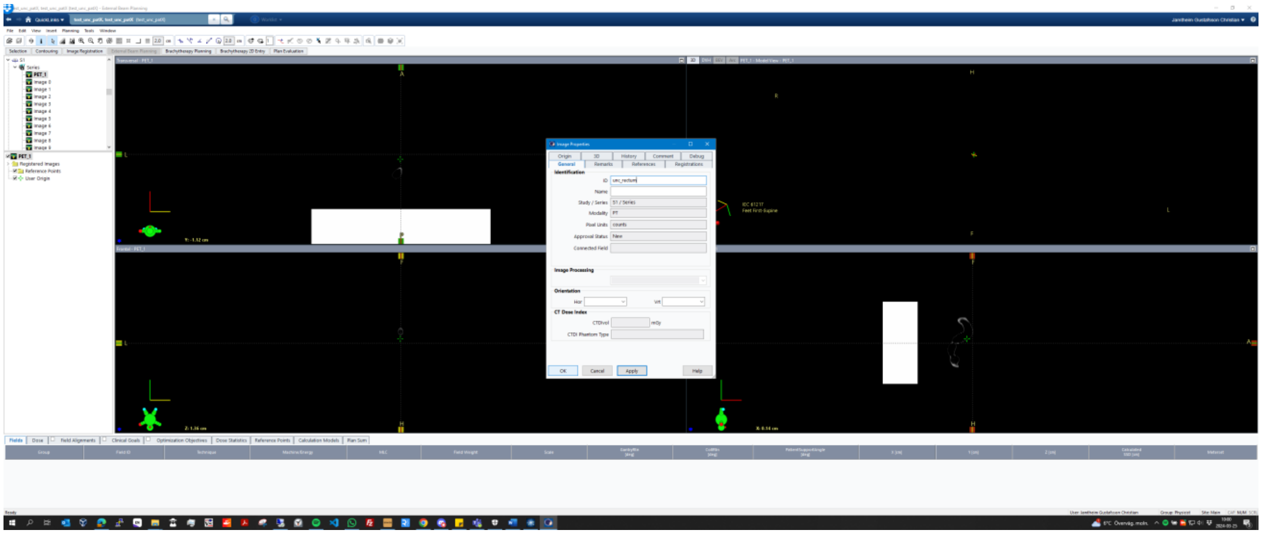

- Rename image volume for CTV to CTV2. (Second step). Similar for rectum struct to Rectum2 (Second step).

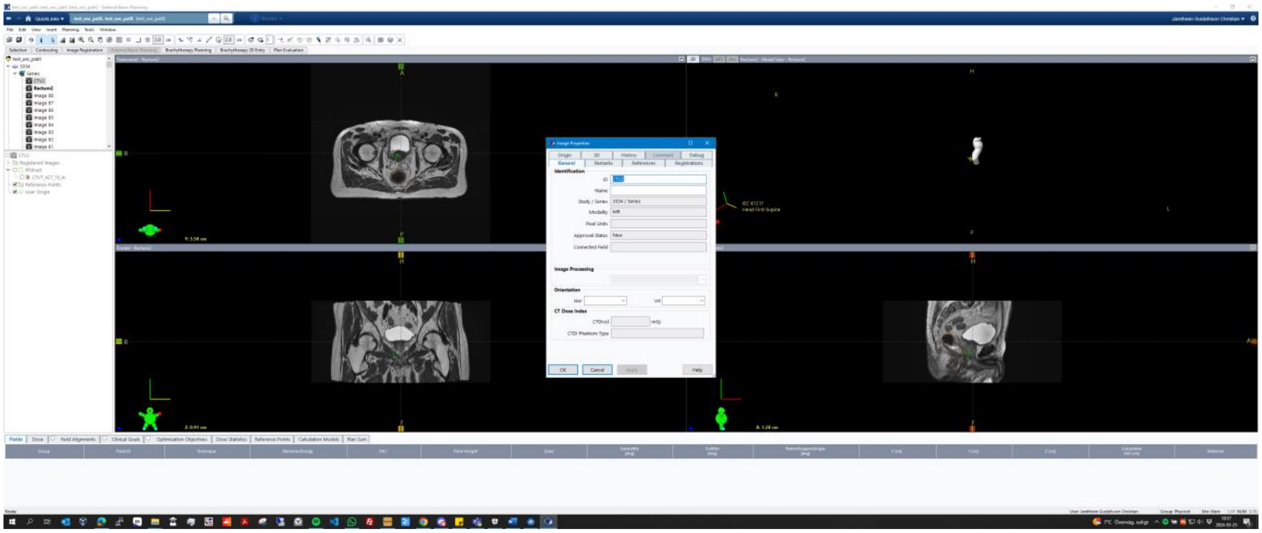

- Save
- Should look like this in contouring

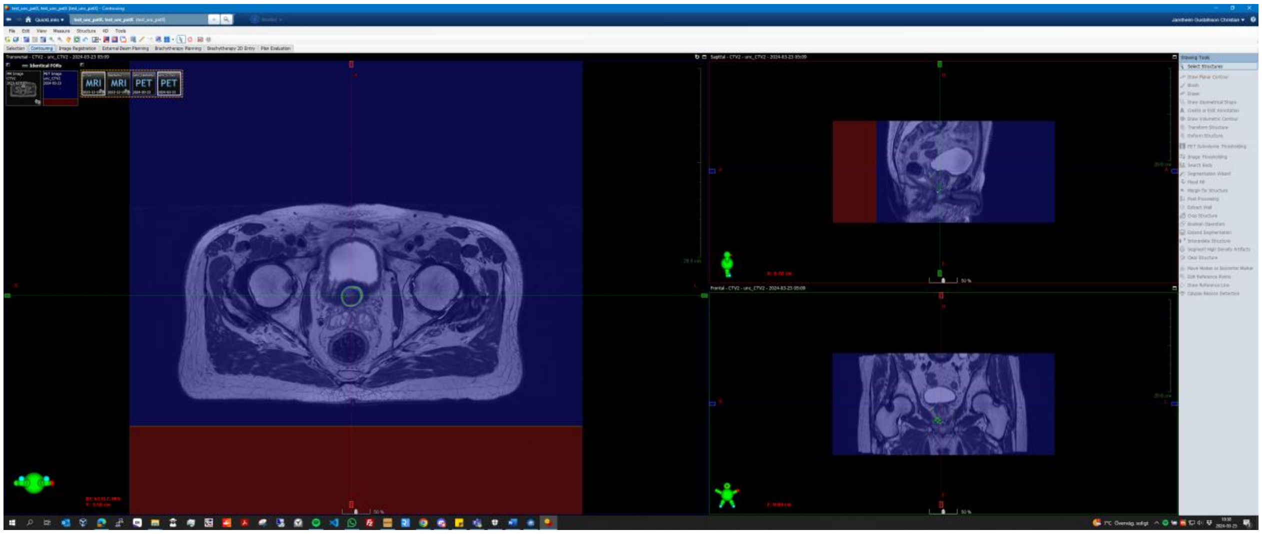

- Point added 2024-04-29. Realization that we needed original unchanged structures from Eclipse as there were potential interpolation errors when comparing ground raw data. Therefore, the two imported structures in this step will need to be directly exported again. No need to export the uncertainty map, it is the same, verified. This is a safety measure so that we can compare the correct things and isolate changes between original, step1 and step 2. So both CTV and rectum needs export directly after import /Christian to Christian
  Copy data to archived study folder
  \\XXX.XXX.XXX.XXX\data2\Christian\MRIOnlyData\inference\observerDataEdited
  /obsN/PatientX/refEclipse/Rectum/
  /obsN/PatientX/refEclipse/CTV/

Observer study instructions - Step 2

Aim: Assess the quality of the AI generated structures and edit them as needed. This is performed with the availability of the uncertainty information to measure the impact it might have on delineation decisions.

* You are about to assess and edit AI generated structures, remember that all such structures are computer generated and may therefore be incorrect and carry uncertainties. In addition to the auto generated structure, we are also displaying information regarding where in the image segmentation these uncertainties may be.

Before you start, answer the question (once only, not per case):

Do you think delineation uncertainty information will provide you with useful info?
(Include this in the Google sheet)

Open the dedicated Google sheet depending on what observer identify you have. It contains assessment questions and a matrix to record finished subjects.

Observer A:

Kommer senare

Observer B:

Kommer senare

Observer C:

Kommer senare

Observer D:

Kommer senare

Observer E:

Kommer senare

Look at the patient matrix in the google sheet. Handle/perform the subjects in the specified order. You will find the full patient pointer at the **subject name + _obsN**). For example, if you are observer A you will find the first patient at "**test_unc_pat4_obsA**".

19	**Subject**	Structures edited/approved	Q1
20			A/B/C/D/E
21	test_unc_pat4		
22	test_unc_pat26		
23	test_unc_pat29		
24	test_unc_pat11		
25	test_unc_pat35		

- Open selected patient in Eclipse and go to contouring module.
- Double check that you loaded the correct patient, as this is observer specific. Patient order is randomized according to the Google sheet.
- It is important to set you settings for displaying a PET image according to this: In the upper menu:

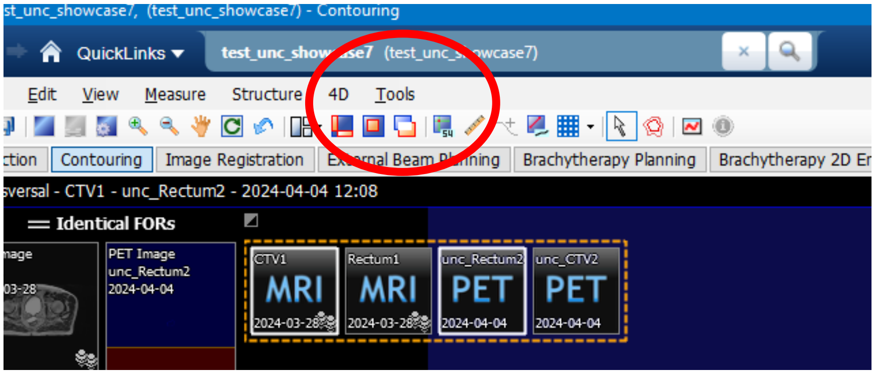

Tools, options, display, PET, PET Rainbow 2D and Pet Rainbow 3D.

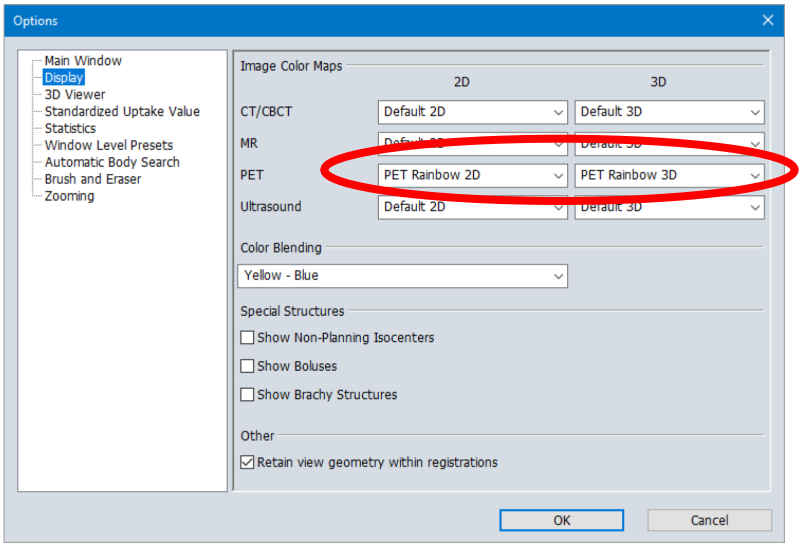

- When patient is loaded it looks like this. Please confirm correct PET display settings for Rainbow color scheme.

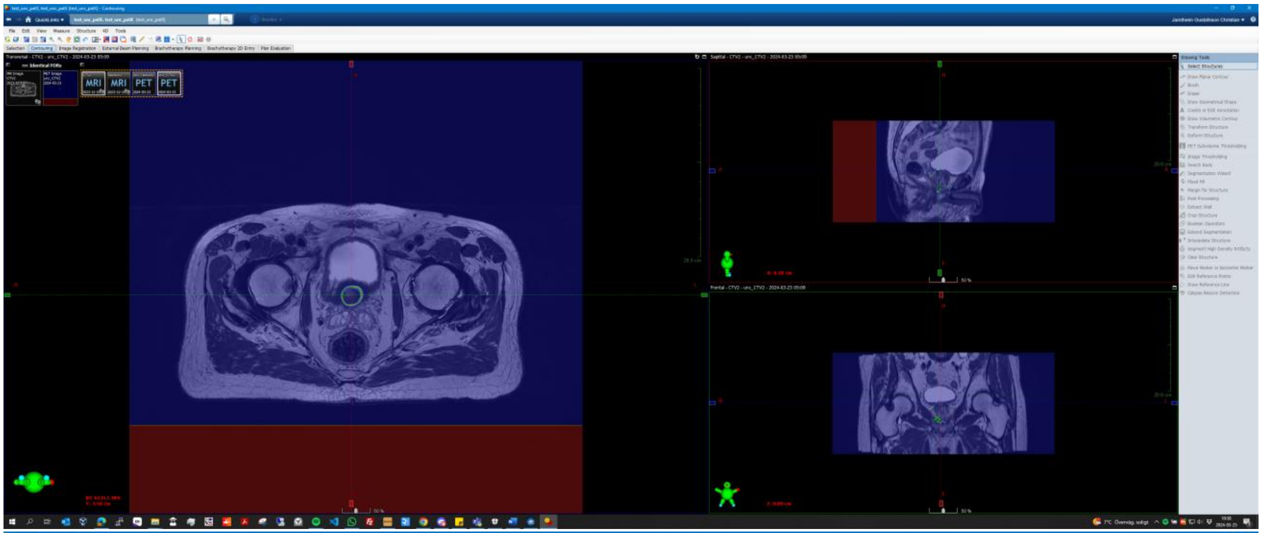

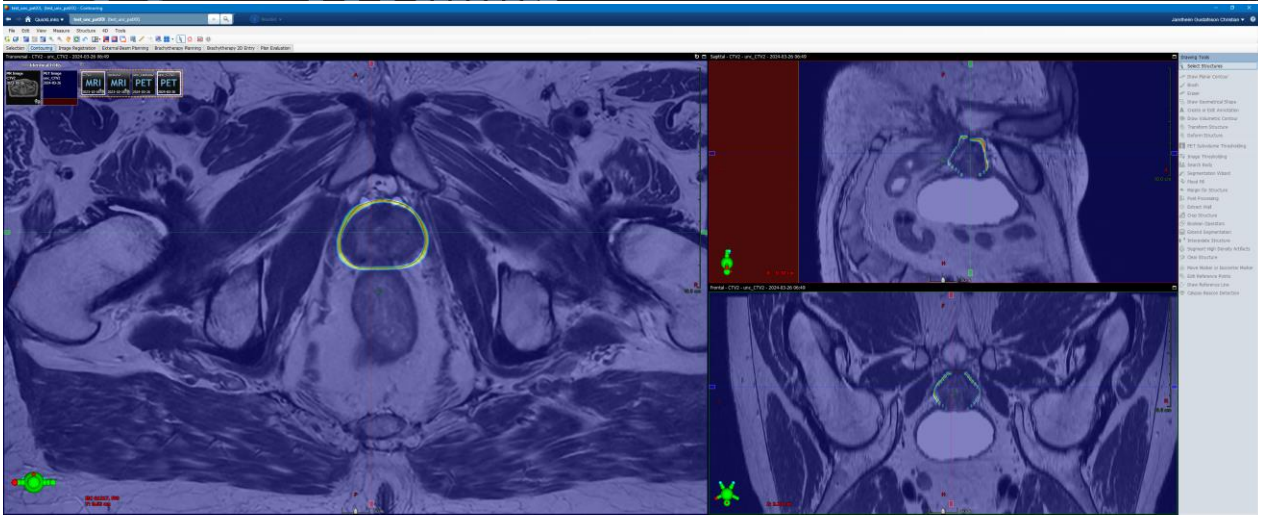

- There are two volumes named CTV2 and Rectum2. These are the same as the unedited ones in Step 1, just named differently. Volumes are identical but have different structures in them.

- Please review questions in the Google sheet and answer question 1 and 2 by providing your answer in the subject matrix under each specific question (Q1 and Q2). Observe the answer alternatives.

- You should blend between the MRI/delineation and uncertainty map and it is encouraged to do so. To do this, double click the orange line around the volumes. Select matching pair CTV2/unc_ctv2 or Rectum2/unc_rectum2. Make sure the MRI series and then the PET series is the ones located to the upper left corner:

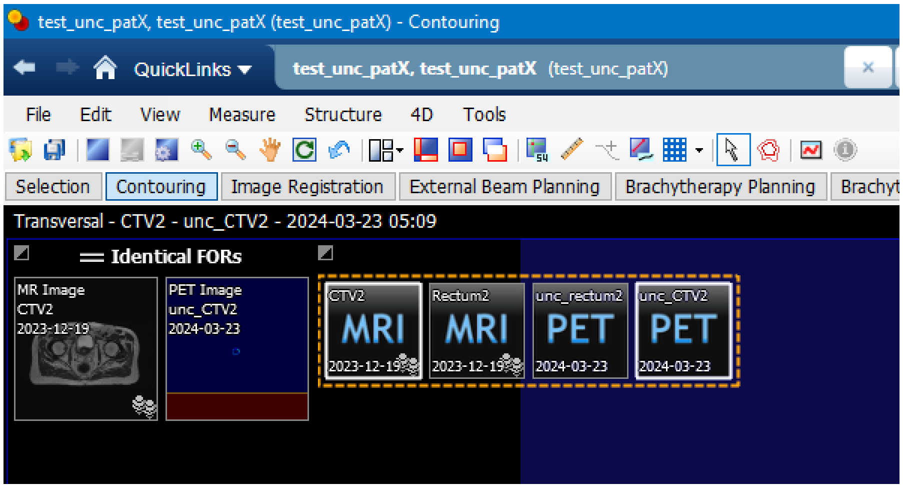

Counts in uncertainty map can be converted to % uncertainty. In the example below we have 3532 counts, this represents 35.32% (a standard deviation of 0.35). The color scale for the uncertainty map represents the delineation standard deviation for each voxel where most blue color means a standard deviation of 0 and most red color means 0.5 in standard deviation, all with respect to the AI model delineation output. Color scale is set to the same for all subjects (0 to 0.5 in standard deviation).

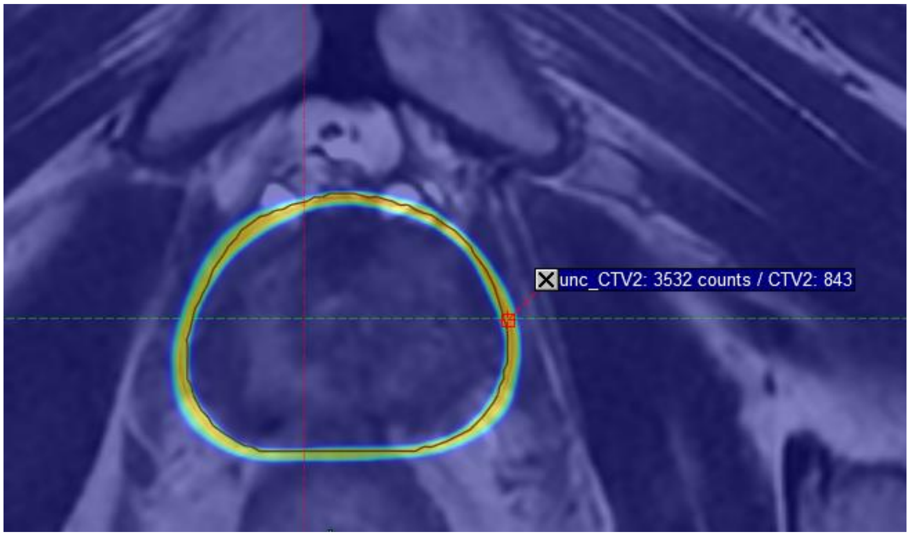

- Perform your edits (if needed) to the structure in the volumes CTV2 and Rectum2 using the assistance of the uncertainty map.

For CTV2 the structure is called CTVT_427_10_AI

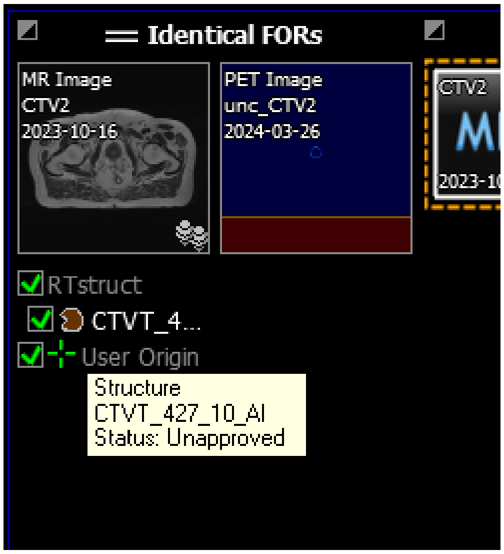

For Rectum2 the structure is called Rectum_10_AI

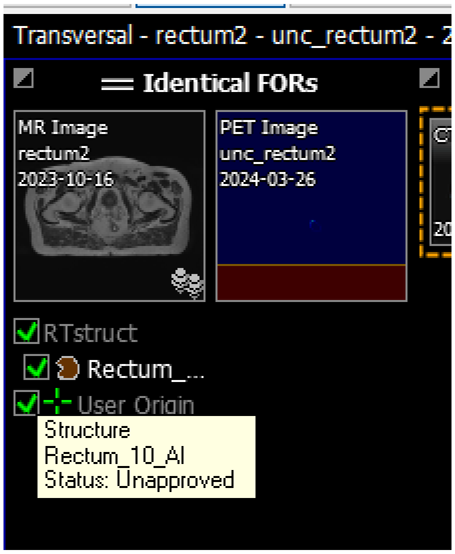

- Answer the question 3 in the Google sheet. Providing your answer in the subject matrix under Q3 CTV and Q3 rectum. Observe the answer alternatives.

- Save patient after edit is finished.

- Mark the patient as complete in the Google sheet by setting a green box in the cell. Make sure you answer all questions and put a green box for all finished tasks. It is appreciated if this can be done after every subject. In that way we can measure time.

After all patients are done according to the patient list in the Google sheet in Step 2, answer the questions once:

Do you think uncertainty has provided you with useful info?
What was the benefits with uncertainty information?
What was the drawbacks with uncertainty information?
What do you think the potential of uncertainty information is?
(Include this in the Google sheet)

Export - Step 2 – Physcis Step

- Open selected patient for correct observer and patient.

- File/Export/ to MRI only DICOM node.

Select only the series with the edited structures

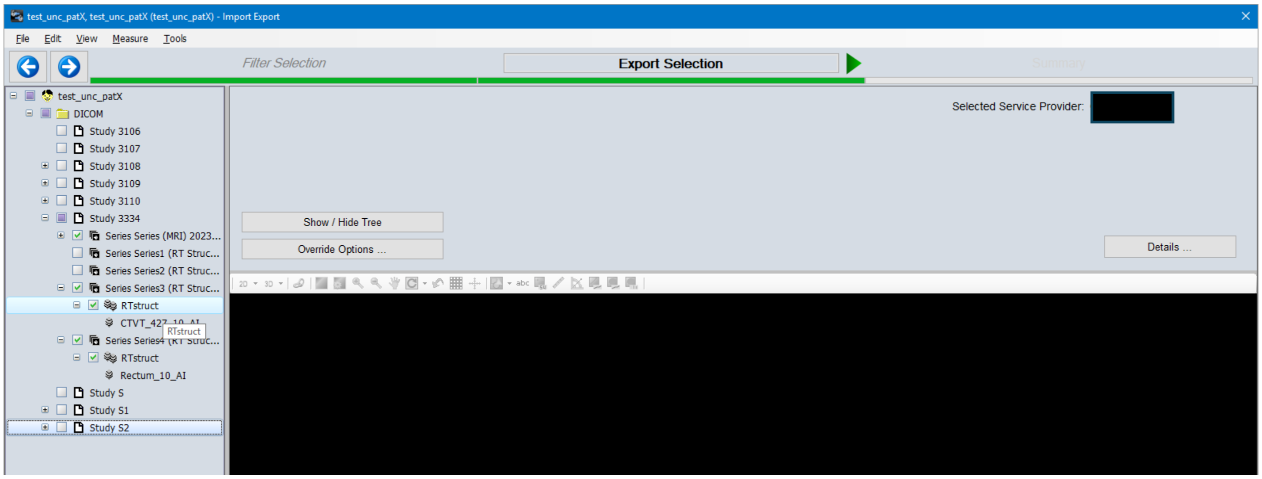

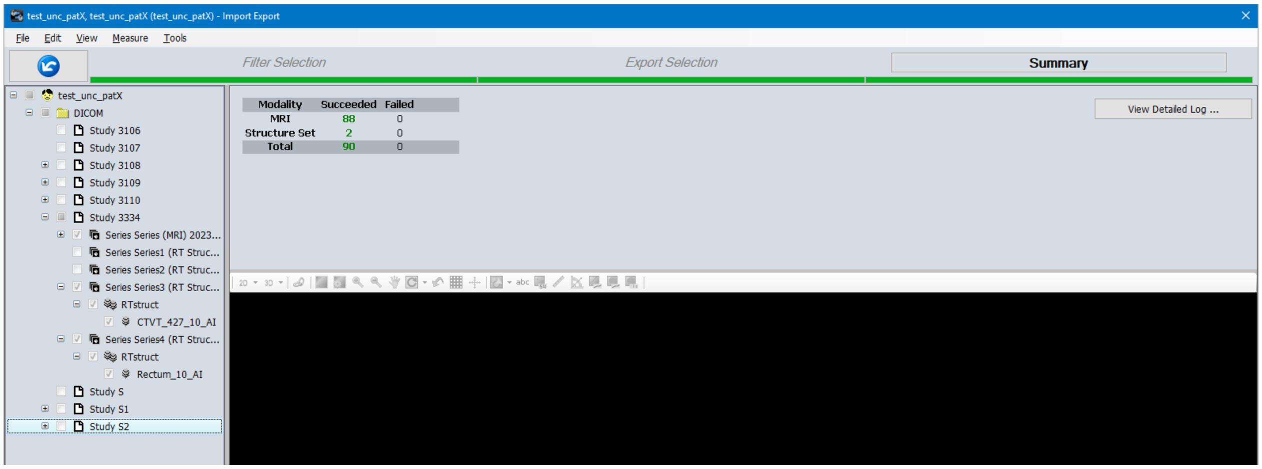

- Check SF archive under RT struct folder. There we will find the edited and exported structures.

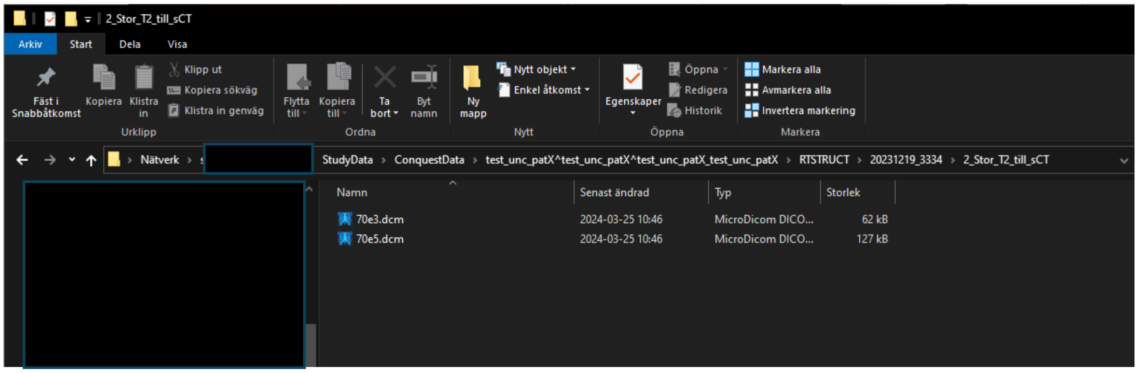

- Copy the data including the MRI. In this case it is \\XXX.XXX.XXX.XXX\StudyData\ConquestData\test_unc_patX^test_unc_patX^test_unc_patX_test_unc_patX.
  Move data to archived study folder
  /obsN/patientX/step2Edits/Rectum2/
  / obsN/patientX/step2Edits/CTV2/
